# The Role of Liquid Biopsy in Gastroenteropancreatic Neuroendocrine Neoplasms

**DOI:** 10.3390/cancers16193349

**Published:** 2024-09-30

**Authors:** Catarina Almeida, Lorenzo Gervaso, Gianmaria Frigè, Francesca Spada, Lavinia Benini, Chiara Alessandra Cella, Luca Mazzarella, Nicola Fazio

**Affiliations:** 1 Division of Gastrointestinal Medical Oncology and Neuroendocrine Tumors, European Institute of Oncology, IEO IRCCS, 20141 Milan, Italy; catarina.lopes.almeida@chsj.min-saude.pt (C.A.); francesca.spada@ieo.it (F.S.); lavinia.benini@ieo.it (L.B.); chiaraalessandra.cella@ieo.it (C.A.C.); luca.mazzarella@ieo.it (L.M.); 2 Department of Medical Oncology, São João University Hospital Center, 4200-319 Porto, Portugal; 3 Laboratory of Translational Oncology, European Institute of Oncology, IEO IRCCS, 20141 Milan, Italy; gianmaria.frige@ieo.it

**Keywords:** liquid biopsy, neuroendocrine neoplasms, circulating tumour DNA, precision medicine, gastroenteropancreatic tract

## Abstract

**Simple Summary:**

Neuroendocrine neoplasms, primarily found in the gastroenteropancreatic tract, are classified as neuroendocrine tumours and neuroendocrine carcinomas, depending on the grade. Liquid biopsy offers a less invasive alternative to tissue biopsy in detecting circulating tumour components in body fluids. This systematic review evaluates liquid biopsy applications in neuroendocrine neoplasms, approaching its various types in different settings, such as diagnosis and characterisation; prognostic and predictive value; monitoring treatment response and prediction of recurrence; and finally, current applications and future perspectives. Overall, liquid biopsy holds potential for managing neuroendocrine neoplasms, and its use should be standardised.

**Abstract:**

Neuroendocrine neoplasms incidence has been increasing, arising the need for precise and early diagnostic tools. Liquid biopsy (LB) offers a less invasive alternative to tissue biopsy, providing real-time molecular information from circulating tumour components in body fluids. The aim of this review is to analyse the current evidence concerning LB in NENs and its role in clinical practice. We conducted a systematic review in July 2024 focusing on LB applications in NENs, including circulating tumour cells (CTCs), circulating tumour DNA (ctDNA), micro RNA (miRNA), messenger RNA (mRNA) and extracellular vesicles. Sixty-five relevant articles were analysed. The LB showed potential in diagnosing and monitoring NENs. While CTCs face limitations due to low shedding, ctDNA provides valuable information on high-grade neoplasms. MiRNA and mRNA (e.g., the NETest) offer high sensitivity and specificity for diagnosis and prognosis, outperforming traditional markers like chromogranin A. The LB has significant potential for NEN diagnosis and monitoring but lacks widespread clinical integration due to limited prospective studies and guidelines, requiring further validation. Advances in sequencing technologies may enhance the clinical utility of LB in NENs. Future research should focus on refining LB methods, standardising protocols and exploring applications in high-grade NENs.

## 1. Introduction

Neuroendocrine neoplasms (NENs) represent a group of heterogeneous neoplasms, for primary site, clinical presentation and response to treatment [1]. They develop from cells of the neuroendocrine diffuse system, potentially arising in many organs but more commonly in the gastroenteropancreatic (GEP) tract. Neuroendocrine neoplasms (NENs) are defined as neuroendocrine tumours (NETs) and neuroendocrine carcinomas (NECs), subdivided into well or poorly differentiated, respectively, based on their morphological characteristics. Regarding their clinical behaviour, NETs usually tend to have a relatively indolent course and, therefore, a better prognosis compared with NEC, even though survival differs based on the Ki67 proliferation index [2].

With the increasing incidence of cancer globally, the demand for precise and early screening tools has grown in order to increase diagnostic, prognostic and predictive abilities in this context. The development of a “precision-medicine approach” led to a massive improvement in oncology, tending to tailor the treatment to the molecular profile of each patient. The gold standard for obtaining molecular information is tissue biopsy. Nonetheless, it has some disadvantages—specifically, its invasive nature, lack of accessibility in some anatomical sites and some biopsy/surgery-related risks such as bleeding, pneumothorax, infection or damage of healthy tissue. Moreover, tissue samples usually represent a baseline assessment of the tumours, not taking into account potential molecular modifications coming from anti-cancer treatments. Therefore, liquid biopsy (LB) has emerged as a tool potentially able to fill this gap since it is much less invasive, implying only a simple venous puncture. Furthermore, it allows a longitudinal and dynamic assessment with repeated and consecutive evaluations, if necessary. It has been best studied in blood, although it can be performed in other body fluids such as urine or liquor. Its mechanism is based on the detection of circulating tumour components, such as circulating tumour cells (CTCs), circulating tumour DNA (ctDNA), circulating non-coding RNA, extracellular vesicles (EVs) and tumour-educated platelets (TEP) [3]. Among its limitations are poor accessibility to some tumour locations, like the brain; reduced ctDNA shedding of some neoplasms and from specific metastatic site into the bloodstream, causing a decreased sensitivity; and, currently, the absence of a standardised method of detection [4]. In clinical contexts, LB is a promising tool with growing and high-quality results in some solid tumours, especially colorectal cancer. It embodies one of the best clinical settings for LB determination, particularly for early detection, treatment response evaluation, tumour response monitoring, therapeutic resistance and prediction of recurrence [5]. However, the role of LB in NENs is still not well defined, although several circulating biomarkers have been investigated in gastroenteropancreatic neuroendocrine neoplasms (GEP-NENs).

The aim of this systematic review is to critically analyse the current evidence about LB in NENs and to understand if and how LB could fulfil a role in future clinical practice.

## 2. Materials and Methods

**Figure 1:**
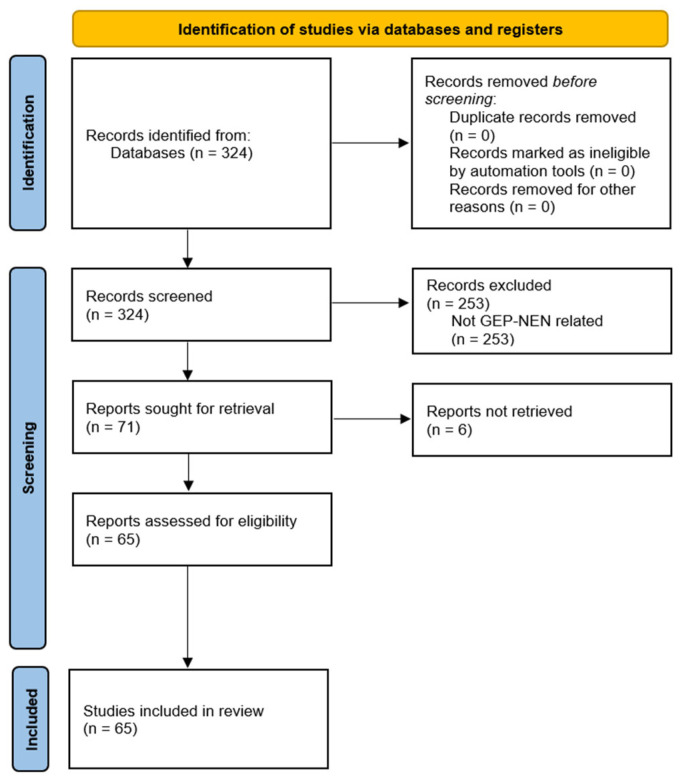
PRISMA flow diagram of the systematic review.

We carried out a review of the literature in July 2024, using the PubMed database, to analyse the available bibliography regarding the application of LB in NENs. Results were restricted to English language, with no limitation about publication type and date. Starting from a broad search strategy using the terms “liquid biopsy” and “neuroendocrine tumours”, or “NEN” or “NEC”, we found a total of 324 articles, without duplicates. After screening all the abstracts, we excluded (1) results that did not address LB in NENs and (2) articles referring exclusively to Merkel cell carcinomas, paragangliomas, pheochromocytomas and pulmonary carcinoid tumours. A total of 65 full-text articles were gathered, including systematic reviews, narrative reviews, retrospective and prospective studies and a single case report (Figure 1). The systematic review followed the recommendations of the Preferred Reporting Items for Systematic Reviews and Meta-Analyses (PRISMA). The protocol has not been registered.

## 3. Results

### 3.1. Types of Liquid Biopsies

#### 3.1.1. Circulating Tumour Cells (CTCs)

Circulating tumour cells were first described by Ashworth in 1869 when he discovered tumour-like cells in the bloodstream and interpreted them as potential metastatic precursors [6]. Some specific neoplasms shed tumour cells into the bloodstream, and they can be differentiated from normal cells through their size and the expression of epithelial adhesion molecules (EpCAMs) [7]. These molecules allow the detection of CTC through the CellSearch^®^ platform, which is approved by the Food and Drug Administration as the detection system for prognostic monitoring of metastatic breast, colorectal and prostate cancers. However, this detection method is considered insufficient and quite limited due to the tumours’ heterogeneity and the possibility of low shedding. Moreover, its clinical value is still debatable. Despite their scarce role in prognosis and diagnosis, they have been shown to have a predictive value about progressive disease, although they should be well correlated with the clinical and pathological results for the assessment of cancer recurrence and metastasis [8]. For midgut NETs, the presence of baseline CTCs (that is, one or more cells) did not demonstrate an improvement in diagnosis, due to its low sensitivity; while limited to small intestine NETs (SI-NETs), its role could be circumscribed to detection of disease progression [9]. Some limitations of this method are the incapacity to identify all tumour cells and the uncertainty about specific cut-off values. Thus, CTCs have not yet been implemented in daily clinical practice and their applicability may be limited to assess genomic changes of the tumour and possibly response to treatments [9,10].

#### 3.1.2. Circulating Tumour DNA (ctDNA)

Circulating tumour DNA makes up only 0.1–10% of the total circulating cell-free DNA (cfDNA) (which can also be detected in healthy individuals). However, it greatly varies depending on tumour histology, tumour burden, disease stage and response to treatment [11]. At present, ctDNA analysis is mostly based on the identification of somatic mutations, either through large sequencing panels (tumour-agnostic) or by individualised assays that detect patient-specific mutations previously identified through high-throughput sequencing of the primary tumour (tumour-informed). The research on this method concerning other carcinomas, mainly non-neuroendocrine of the gastrointestinal (GI) tract, is extensive and already has an active role in clinical practice [5,12,13]. Some of the limitations of the use of ctDNA in NENs are the scarcity of known mutations to detect and the variable amount of released tumour DNA. Some tumour features promote ctDNA detection, such as hepatic metastases, necrosis and high proliferative index. These are more characteristic of NECs, inferring that there could be space for a correlation between high-grade neoplasms and a greater amount of ctDNA [10]. By contrast, NETs, due to their more indolent behaviour, have less ctDNA shedding and are not even detectable in many cases. More recent technologies are exploiting other properties of ctDNA that can be assessed without relying on the presence of somatic mutations, such as evaluating ctDNA fragment length or alterations in methylation [14].

CtDNA and non-malignant cfDNA are released from cells through different biological processes. Since the dominant process for cancer is apoptosis, ctDNA fragments tend to be shorter and more evenly distributed than non-malignant cfDNA, and this property can be leveraged to increase the signal-to-noise ratio in ctDNA analysis. Additionally, epigenetic differences between cancer and noncancer DNA such as DNA methylation are maintained in ctDNA and can also be exploited for non-invasive cancer detection [15].

#### 3.1.3. Micro RNA (miRNA)

Micro RNA belongs to the category of small (with an average of 22 nucleotides in length) non-coding RNAs. Altered miRNA levels are associated with cardiovascular, gastrointestinal, neoplastic and renal diseases. In cancer, miRNA promotes angiogenesis, cell metabolism and metastases. Although widely studied in other tumours, such as ovarian, cervical and breast cancers, there are very few data regarding GEP-NENs [10,16]. One study concluded that a combination of four types of miRNA (miR-125b-5p, miR-362-5p, miR-425-5p and miR-500a-5p) was able to distinguish SI-NETs from healthy individuals [17]. Combinations of multiple miRNAs will likely be needed to identify NENs and assess specific features such as response to treatment and disease recurrence. It is unknown if other parameters, such as treatment with somatostatin analogues, affect the levels of miRNA [17].

#### 3.1.4. Messenger RNA (mRNA)

The epitome of mRNA evaluation is the NETest. This assay was developed by Modlin et al. and the first results were published in 2013, providing a significantly higher accuracy compared to blood chromogranin A (CgA) in NETs diagnosis [18]. Starting from an EDTA peripheral blood sample, a two-step protocol with mRNA isolation, complementary DNA (cDNA) production and real-time polymerase chain reaction (RT-PCR) was performed, assessing the level of expression of 51 neuroendocrine specific genes involved in NEN oncogenesis [19]. The results are expressed as a score, representing the activity index, which ranges from 0% to 100%. The most used cut-off value is 20%. Below 20% would indicate a healthy individual, a percentage between 20 and 40% shows a stable disease and over 40% would suggest a progression of the disease. This test is potentially useful for the diagnosis, prediction of recurrence, prognosis and monitoring the treatment response of GEP-NENs. It has been reported to be highly sensitive [20,21]. Moreover, an advantage over blood CgA is the reliability of results, which are not affected by gastritis or proton pump inhibitor intake [22]. As for its limitations, in theory, the degradation of extracellular circulating mRNA could result in false-negative results by degradation of target genes mRNA whereas false positive results could depend on the degradation of housekeeping genes’ mRNA [23]. Moreover, it should be considered that somatic mutations in non-tumour cells can also produce false-positive results with the use of high-sensitivity platforms. Subsequent studies have questioned its specificity over non-neuroendocrine neoplasms [24].

#### 3.1.5. Extracellular Vesicles

Extracellular vesicles include several components such as proteins, DNA, miRNA and mRNA. After their release, they can either stay close to their tissue of origin or migrate through body fluids, and may therefore be isolated [12]. This is performed through ultracentrifugation, although its quantification is quite challenging. A study found that some proteins are expressed simultaneously in pancreatic cancers, pancreatic NENs (Pan-NENs) and other diseases such as chronic pancreatitis; however, some of them were significantly higher in pancreatic cancer [25].

### 3.2. Potential Applications in Clinical Practice

There are several circulating biomarkers investigated in GEP-NENs, divided into two categories: monoanalyte and multianalyte markers. The former include specific molecules like RNA, a protein or a gene. When NENs are functioning, they secrete hormones, such as insulin, glucagon, vasoactive intestinal peptide or serotonin, causing specific clinical syndromes. These can be used for diagnosis and can be monitored as specific monoanalyte biomarkers. On the other hand, some examples of non-specific monoanalyte markers are CgA, neuron-specific enolase and pancreatic polypeptide, which are analogously detected in the blood [6]. Their biggest handicaps are low sensitivity and specificity and the absence of regulatory measures and means of detection. Therefore, the need for multianalyte tests increased, identifying multiple genomic regulators. Hereafter, we will address the different types of LB in GEP-NENs, which are summarised in Table 1.

#### 3.2.1. Diagnosis and Characterisation

As a diagnostic tool, the NETest had an accuracy of 95–96%, a sensitivity of 89.4–94.4% and a specificity of 95.4–98.7% [21]. It may detect misdiagnosed cases with a safety margin due to its sensitivity; in addition, it also excludes healthy subjects, since the probability to have the disease with a result below the lower cut-off is extremely rare. A case–control study by Malczewska et al. analysed the concordance between NETest and the combined use of imaging plus histology data, comparing patients with NEN, other types of tumours and healthy individuals. There was a 100% agreement between imaging/histology and a positive NETest in Pan-NENs, duodenal NENs (D-NENs) and gastric NENs (G-NENs), while the concordance was lower for SI-NENs and rectal NENs (R-NENs) [39].

The NETest has been reported as superior to blood CgA in terms of sensitivity/specificity in three different prospective case–control studies, evaluating patients with NENs and control groups [20,40,41,42]. Of course, it should be considered that blood CgA is a non-specific marker; therefore, its role in diagnosis of NEN is quite relative. The value of NETest as a screening test for GEP-NENs is poor due to its low specificity. However, it showed superior sensitivity over blood CgA in detecting recurrences during follow-up, as well as in detecting minimal residual disease after radical surgery [23]. Al-Toubah et al. investigated the sensitivity and specificity of the NETest in metastatic GEP and lung NETs, comparing the scores between these patients, non-NEN GI malignancies and healthy individuals. The defined a lower limit of NETest of 13% for disease detection, although the broadly used 20% cut-off was also analysed. The sensitivity was 98% for both cut-offs, with a single false-negative case. With the 20% cut-off, the specificity was 85%, from 100% in healthy individuals to 67% among other malignancies. Therefore, they concluded that the sensitivity of the NETest was outstandingly high in metastatic NET as well as the specificity among healthy individuals with a 20% cut-off. Conversely, the specificity was relatively low in patients with other malignancies [24]. One potential reason may be related to some parameters of the tool, like proliferation and metabolism transcripts, which are not exclusive to NETs. Specifically for NENs, it seems that the role of this test in detecting macro- or microscopic residual disease is higher than reported in previous studies [42].

Boons et al. presented the first longitudinal study in Belgium on patients with metastatic GEP and lung neuroendocrine neoplasms (NENs) treated with everolimus, in which they estimated circulating tumour DNA (ctDNA) by assessing copy number alterations derived from cfDNA through shallow whole-genome sequencing compared to healthy individuals used as reference samples. cfDNA was extracted from the plasma, and ctDNA was detected in 30% of the samples and 44% of the patients; all controls were ctDNA negative. Positive ctDNA was associated with higher histopathological grades, primary tumour location (mostly pancreas), higher CgA, worse OS and worse progression-free survival (PFS) [28]. A study conducted by the Nordic NEN group evaluated the sequencing of 76 genes in ctDNA in 50 patients with advanced GEP-NECs and compared them to the ones found previously in solid biopsies. Liquid/solid concordance rate was 71%, whereas 29% of the alterations were exclusively found in LB. The concordance was higher in patients with hepatic metastases, probably due to increased shedding, and it was lower in oesophageal cases [29]. Similarly, Zakka et al. retrospectively studied the role of NGS testing to characterise alterations in the genomic landscape. From 338 plasma samples of patients with GEP and lung NENs, genomic alterations were observed in 87% of cases. The most commonly altered genes were *TP53* (52%), *KRAS* (22%), *EGFR* (12%), *PIK3CA* (11%), *BRAF* (10%), *MYC* (10%), *CCNE1* (10%), *CDK6* (8%), *RB1* (7%), *NF1* (7%), *MET* (7%), *FGFR1* (7%), *APC* (7%), *ERBB2* (6%) and *PTEN* (5%). The analysis of ctDNA could be useful for early detection of recurrent disease and to investigate genomic alterations that may eventually have therapeutic targets [30]. Lastly, the role of tumour genomic profiling through ctDNA has been investigated in 45 patients with NETs. There were significantly more alterations identified in non-well-differentiated NETs than in well-differentiated ones. In this last group, no currently druggable molecular alterations have been identified, therefore not resulting in changes in the therapeutic management. These results could probably be related to the lower prevalence of significant alterations in well-differentiated NETs rather than to technical limits in ctDNA detection [31].

The advent of novel sequencing techniques such as Oxford Nanopore, based on single-molecule sequencing and real-time analysis, will allow multi-omic characterisation in a single assay, addressing the lack of limited material for molecular analysis. Assessing single-nucleotide variants (SNVs), structural variants (SVs), cfDNA methylation and fragmentomic patterns together, used as surrogates for gene expression, will enable comprehensive tumour characterisation from circulating DNA. Specifically, the study of fragmentation patterns (also known as fragmentomics) is currently a developing area of biomarker research. Given this lack of information concerning cancer DNA fragments in the plasma sample, it is necessary to sequence them at high depth coverages to obtain enough material and, hence, achieve early cancer detection.

#### 3.2.2. Prognostic and Predictive Value

In a recent sub-analysis of a prospective study, a cohort of GEP-NET patients was managed with a watch and wait strategy. In those cases, the concordance between a low NETest and stability of disease was 100%, while a high NETest was associated with a change in the clinical management in 83% of patients [32]. In a study of 34 patients with advanced GEP-NETs followed for a median of 4 years, the NETest was the only baseline variable (Cox modelling) associated with PFS (hazard ratio = 1.022, 95% confidence interval = 1.005–1.04; *p* < 0.012) [33]. Kidd et al. described the expansion of 14 components of the NETest omic cluster, including apoptome, epigenome, growth factor signalome, proliferome, secretome and SSTRome. The aim was to understand which of these omics were overexpressed in NETs compared to controls and to determine if it changed the prognostic accuracy. Thirteen (93%) were differentially regulated in NETs: twelve upregulated, one (apoptome) downregulated and one (inflammasome) without any difference to controls. Assessing differences in disease progression, seven parameters were increased in patients with disease progression—proliferome, NEDome, SSTRome, metastasome, neurome, epigenome and fibrosome—categorised as canonical omes of disease progression. Patients with disease progression showed significantly elevated expression of these seven omes (*p* < 0.0001), strengthening the rationale that a specific genomic signature for disease progression could be identified by the NETest, with prognostic implications [34].

Regarding the predictive value to systemic treatment, Cwikła et al. evaluated whether the NETest predicted response to somatostatin analogues (SSAs). The test was positive in every patient and its score was significantly lower in stable disease than in progressive disease, with a significant association concerning treatment response (*p* = 0.002) [35]. Three prospective studies enrolling patients with GEP and lung NETs evaluated treatment response to peptide receptor radionuclide therapy (PRRT). Two of them compared the PRRT prediction quotient (PPQ), based on Ki67 percentage and NETest, in 67 and 122 patients treated with 177Lu-DOTATATE. The PPQ accurately predicted response in 96% and 97% of patients, in both studies. Moreover, responders had a significant decrease in NETest scores (−37 ± 44%) at follow-up, while non-responders had an increased score (76 ± 56%) at progression [36,37]. The third study compared the monitoring of treatment response between NETest and CgA in 54 patients, with a prediction of response of 89% for NETest and 24% for CgA [50]. Although PRRT is a safe treatment option, it can cause renal and bone marrow toxicity, especially in long-term survivors. Thus, it is crucial to have the possibility to predict responders through the identification of transcript profiles [22,38].

The cfDNA concentration was shown to be significantly higher in metastatic NEN, along with increased fragmentation and hypomethylation, when compared to healthy controls. Therefore, this combination of cfDNA characteristics is a strong and sensitive prognostic marker for tumour burden and disease progression [43].

#### 3.2.3. Monitoring Treatment Response and Prediction of Recurrence

Due to its low invasiveness and easy feasibility, blood LB would be the ideal tool for evaluating tumour response in GEP-NEN patients to both systemic and locoregional treatments. As a type 2 biomarker (surrogate endpoint), in a systematic review by Oberg et al., the NETest was reported with an accuracy of 93.7–97.4%, sensitivity of 88–90.1% and specificity of 99.6–99.7% [21]. This makes it an excellent biomarker for predicting response to treatment: an NETest score of >40% would identify treatment failure and a score of ≤40% would demonstrate treatment efficacy. Regarding surgical treatment, a prospective study evaluated NETest in Pan-NET patients that underwent radical surgery on postoperative days 1, 5 and 30. The preoperative score was high in every patient and the NETest scores significantly decreased after radical resection, suggesting a fair early assessment of surgical efficacy (*p* = 0.006) [44]. Similarly, another study showed a decreased level of NETest after resection compared to baseline. Interestingly, around 30% of patients had persistently elevated values and, after 18 months, 81% of them had radiologic recurrence, correlating with residual disease after surgery [45].

Van Treijen and colleagues prospectively evaluated NETest scores in GEP-NETs after surgery, concluding that it was an independent predictor of disease progression and correctly classified 82% of the cases [46]. Thirteen SI-NENs patients treated with curative intent remained without evidence of disease during follow-up, consistent with lower scores. On the opposite, patients with synchronous oligometastatic disease had an elevated NETest score post-surgery and eventually experienced relapse [47].

Modlin et al. performed a prospective multicentre evaluation of 103 patients with GEP and lung NETs submitted to surgical resection, all with elevated NETest scores before surgery. The patients were divided into groups considering post-surgery residual disease: no residual tumour (R0), microscopic residual tumour (R1) and macroscopic residual tumour (R2). On day 30 after surgery, in the R0 group, the NETest score decreased from 59 ± 28% to 26 ± 23% (*p* < 0.0001). Conversely, in all patients whose score remained high, radiological recurrence occurred during follow-up. In the R1/R2 group, even though there was a decrease in the NETest score, 100% remained elevated in day 30. This study showed a 94% concordance for predicting relapse and a reduction in costs of 42% by using NETest instead of imaging [48]. A study from Treijen et al. compared serial samples of NETest with radiologic findings in 132 patients with GEP-NET, managed with a watch and wait strategy, for four years. In the non-treated patients who had disease progression, 82% of the NETest scores were consistently high, while low scores were associated with a more indolent course of the disease [49]. Lastly, Genç et al. evaluated the role of NETest for early detection of recurrence during follow-up in Pan-NETs treated with surgery. NETest scores were statistically higher in R0 patients who experienced recurrence (56 ± 8%) compared with R0 and even R1 patients without recurrence (39 ± 6% and 28 ± 6%, respectively) [27].

With respect to CTCs, Childs et al. compared single-cell profiling in CTCs with tumour tissue through next-generation sequencing (NGS) in seven patients with metastatic NEN. The copy number variation reported recurrent chromosomal alterations in NENs, including loss of chromosome 18. There were also CTCs with distinct clonal lineages and genomic heterogeneity, which could lead to important advances in identifying therapeutic targets [51].

## 4. Discussion

### Current Applications and Future Perspectives

Liquid biopsy does not have a defined role in clinical practice in patients with GEP-NEN yet. Among the various types of LB studied in GEP-NENs, the mRNA-based NETest represents the most frequently investigated in several different clinical settings, including diagnosis, prognosis, evaluation of response to treatment and prediction of recurrence. Clinical investigation with ctDNA is still limited.

Despite the relatively high number of published papers addressing the clinical value of the NETest, it has not been included in any guidelines to date. Several reasons could be supposed, including the low level of evidence and heterogeneity of the studies—being mainly retrospective—and/or the fact that no major prospective clinical trials on NEN investigated the NETest value. Furthermore, as stated in the meta-analysis published in the Annals of Oncology, the “principal clinical utility of the NETest is to monitor disease progression and assess prognosis”. This could have been considered not so relevant information given that other well-consolidated tools are already validated to the same purposes [21]. On the other hand, NETest could be utilised occasionally in very select cases together with other parameters usually utilised for decision making, especially in clinical settings where minimal residual disease is hypothesised, but always with a discussion within a NEN-dedicated multidisciplinary team.

With regards to ctDNA, it is important to consider that well-differentiated NETs are less likely to have targetable alterations that may impact patient management. Nevertheless, there are some mutations that are uniquely detected in liquid biopsies. As it is difficult to perform both in real life, liquid biopsies could be used if the solid biopsy is unobtainable or in cases where no targetable mutations are detected in the solid tumour biopsy.

Recently, there has been research around the use of cancer-specific DNA methylation signals in order to overcome the existing limitations of conventional mutation-based liquid biopsy testing [52]. A recent paper demonstrated the potential of targeted cell-free DNA (cfDNA) methylation analysis as a non-invasive method for detecting neuroendocrine prostate cancer (NEPC). By identifying specific methylation patterns unique to NEPC and quantifying the tumour fraction in the bloodstream, the study suggests that this approach could significantly improve early diagnosis and treatment outcomes for this cancer type [53]. An additional study indicated that the quantification and integrity of cfDNA, combined with its hypomethylation status and fragment length patterns, are reliable indicators of early detection of metastatic disease prognosis and treatment response in patients with advanced NENs [43].

Nanopore technology has been growing, providing sequencing of any DNA or RNA fragment length, from short to ultra-long, in real-time analysis. It allows the identification of genomic aberrations, namely, in circulating tumour samples, such as single-nucleotide variants, structural variants and base modifications. In a recent study, an optimised workflow for Oxford Nanopore-based cfDNA methylation, combined with the use of machine learning models on signals from nanopore sequencing data, has enabled the detection of cancer-specific methylation profiles. The extracted DNA underwent nanopore sequencing with methylation profiling. For each individual read, it was measured how similar its methylation profile was to another reference sample’s methylation profile. This approach has the potential to impact liquid biopsy diagnostics for cancer detection and characterisation. Additionally, the study demonstrated the potential clinical utility of this method by monitoring cfDNA methylation dynamics throughout the treatment of various cancer patients, including one with metastatic pancreatic neuroendocrine carcinoma. These patients were observed longitudinally, with their cfDNA methylation profiles analysed to establish correlations with clinical outcomes such as tumour response and recurrence [54].

Another ongoing issue is the fact that molecular blood biomarkers have proven their value in lower-grade NETs but have not been much studied in high-grade GEP-NENs. The histological distinction between NEC, grade 3 NET and mixed neuroendocrine–non-neuroendocrine neoplasms (MiNEN) could be difficult. A recent prospective study performed the NETest in high-grade GEP-NENs before first-line chemotherapy and concluded that the test was almost always elevated in NECs, especially large-cell NECs. Regarding grade 3 NETs and MiNENs, the test was less frequently elevated; as for adenocarcinomas, the test was always normal. Although a high level of NETest corresponded to a shorter survival in this population, this study did not show a role for NETest as a tool to distinguish between NEN and non-NEN or between NET G3 and NEC. However, this could be due to the very low number of cases, especially for some subgroups, and the heterogeneity of the population; therefore, further studies are warranted to investigate this question [55]. Other types of liquid biopsy could be utilised to this purpose, as observed by Knappsnog and colleagues using NGS Avenio ctDNA Expanded (version 2.0.0), which covers 77 cancer genes [29].

We have an ongoing project that aims to develop a liquid biopsy assay based on the combination of methylated DNA and fragmentomics analysis, which will be able to detect circulating tumour DNA of NE derivation. We will take advantage of the novel Oxford Nanopore sequencing technology, exploiting its capability for simultaneous analysis of DNA sequence and methylation. Through the comparison of newly generated epi-transcriptomic landscapes maps of GEP-NENs from cell lines and primary surgical specimens with cfDNA methylation and fragmentomics signals, we will assess the cell of origin. This approach will allow the discrimination of signals specifically generated within NET cells from noise due to surrounding nontumoral or exocrine tumoral cells. This will enable the non-invasive diagnosis and monitoring of GEP-MiNENs, addressing a relevant, unmet clinical need.

## 5. Conclusions

In terms of diagnosis, LB could be useful in case of inconclusive or inaccessible tissue biopsy, or even in contradictory cases, such as if the imaging methods are negative but the clinical suspicion remains high. Although the evidence is still not strong enough to use these tools as a screening method for GEP-NENs, the NETest in particular could be used as a disease marker to detect recurrences during follow-up as well as to detect post-operative residual disease. As far as prognosis is concerned, prospective studies showed that most patients with a low NETest score remained without evidence of disease, whilst those with higher scores eventually presented disease progression. These studies showed that LB could predict disease recurrence even before imaging techniques do. This could be a game changer in clinical practice if it was incorporated in clinical guidelines, as it would reduce costs in the follow-up of NEN patients.

## Figures and Tables

**Table 1:** Main types of liquid biopsy and their possible applications in clinical practice.

Types of Liquid Biopsy	Main Clinical Applications
Circulating Tumour Cells (CTCs)	Predictive value on disease progression and survival; treatment response [8,9,10,26,27]
Circulating tumour DNA (ctDNA)	Diagnosis and characterisation; predictive value on disease recurrence [5,12,13,28,29,30,31,32,33,34,35,36,37,38]
Micro RNA (miRNA)	Diagnosis and characterisation [17]
Messenger RNA (mRNA)	Diagnosis; prognostic and predictive value on disease recurrence; monitoring treatment response [20,21,39,40,41,42,43,44,45,46,47,48,49]
Extracellular vesicles	Characterisation [25]
